# Diet alters delayed selfing, inbreeding depression, and reproductive senescence in a freshwater snail

**DOI:** 10.1002/ece3.1146

**Published:** 2014-06-22

**Authors:** Josh R Auld, John F Henkel

**Affiliations:** Department of Biology, West Chester University750 S. Church St., West Chester, Pennsylvania, 19383

**Keywords:** Age at first reproduction, aging, caloric restriction, mating system, pulmonate, transgenerational effects, waiting time

## Abstract

Reproductive success is a critical fitness attribute that is directly influenced by resource availability. Here, we investigate the effects of diet-based resource availability on three interrelated aspects of reproductive success: a change in mating system based on mate availability, consequent inbreeding depression, and the deterioration of reproductive efficiency with age (senescence). We employed a factorial experimental design using 22 full-sib families of the hermaphroditic freshwater snail *Physa acuta* to explore these interactions. Individual snails were reared in one of two mate-availability treatments (isolated [selfing] or occasionally paired [outcrossing]) and one of two diet treatments (boiled lettuce or *Spirulina*, an algae that is rich in protein, vitamins, and minerals). *Spirulina*-fed snails initiated reproduction at a 13% earlier age and 7% larger size than lettuce-fed snails. *Spirulina* also resulted in a 30% reduction in the time delay before selfing. Compared to lettuce, a diet of *Spirulina* increased inbreeding depression by 52% for egg hatching rate and 64% for posthatching juvenile survival. Furthermore, *Spirulina* led to a 15-fold increase in the rate of reproductive senescence compared with a diet of lettuce. These transgenerational, interactive effects of diet on inbreeding depression and reproductive senescence are discussed in the context of diet-induced phenotypic plasticity.

## Introduction

Diet-based resource availability is one of the most important environmental factors influencing life history and consequently fitness-related traits. Especially when resources are limiting, organisms face allocation trade-offs that can affect the timing of reproduction, growth, and the amount of resources allocated to reproduction (van Noordwijk and de Jong 1986; Stearns 1992; Roff 2002). Furthermore, consistent with the disposable soma theory, moderate resource limitation (i.e., dietary restriction) has been theoretically and empirically shown to alter life history expression and extend life span (Shanley and Kirkwood 2000; Kirkwood and Shanley 2005; Partridge et al. 2005). Resource availability may also affect the expression of a variety of other fitness attributes such as adaptive phenotypic plasticity, inbreeding depression and the expression of senescence, and the interactions between resource availability, and these important fitness attributes are increasingly attracting the attention of researchers (e.g., Chippindale et al. 1997; Lee et al. 2008; Zajitschek et al. 2012; Harwood et al. 2013).

The initiation of reproduction is one of the most important transitions that an organism undergoes. Early reproduction results in a shorter generation time but can have detrimental consequences on longevity (Stearns 1992; Roff 2002). Individuals may delay the initiation of reproduction for a variety of reasons, such as low food availability or a lack of available/desirable mates. Hermaphroditic organisms that are capable of self-fertilization face an additional option in that they can reproduce without a partner (i.e., by self-fertilization). Previous theoretical (Tsitrone et al. 2003a) and empirical (Tsitrone et al. 2003b; Escobar et al. 2011; Ramm et al. 2012) work has revealed a widespread pattern of delayed selfing by reproductively mature, preferentially outcrossing individuals when mates are absent. By delaying selfing when mates are absent, an individual can reserve resources that can be used to produce higher quality (outcrossed) eggs if a mate becomes available (i.e., the avoidance of inbreeding and its associated costs – inbreeding depression and gamete discounting). By delaying reproduction, individuals reserve the ability to self-fertilize in the complete absence of a mate and therefore can exhibit a “best of both worlds” strategy (Lloyd 1979). As such, the waiting time before selfing represents a form of adaptive life history plasticity in that, in an environment with mates, the best strategy is to outcross and avoid inbreeding depression but in an environment without mates delayed self-fertilization is preferable to no reproduction at all (Lloyd 1979; Auld 2010; Escobar et al. 2011). To date, the effects of dietary restriction on this form of adaptive plasticity have not been evaluated.

It has also been observed that, as theoretically expected, the magnitude of inbreeding depression (i.e., the relative fitness detriment suffered by inbred offspring compared with outbred offspring; Charlesworth and Charlesworth 1987; Johnston and Schoen 1994) is positively related to the time that individuals wait before selfing in the absence of mates (Escobar et al. 2011). That is, when the penalties associated with inbreeding are higher, individuals avoid (i.e., delay) inbreeding to a greater extent. Conversely, when inbreeding depression is weak or absent, individuals do not delay selfing. While it is increasingly recognized that inbreeding depression can change in different environmental conditions (Armbruster and Reed 2005; Cheptou and Donohue 2011), very little is known about how the environment can simultaneously alter inbreeding depression and the pattern of inbreeding avoidance (i.e., delayed selfing). Auld (2010) showed that the presence of predator cues reduced the delay in selfing and the magnitude of inbreeding depression suffered by offspring in the freshwater snail *Physa acuta*. To our knowledge, no previous studies have explored the effect of dietary restriction on this relationship.

When organisms express a waiting time before selfing to, for example, avoid inbreeding depression, an obvious consequence is that reproduction is initiated at a later age. Age itself can affect reproductive success, and the intra-individual deterioration in fitness with age (i.e., senescence) is a widely observed aspect of life history expression in numerous organisms (e.g., Rose 1991; Charmantier et al. 2006; Escobar et al. 2008; Auld et al. 2014). Previous work, primarily on insects, has shown that dietary restriction can alter the aging process, often by slowing actuarial (lifespan) and reproductive senescence (Moya-Laraño 2002; Partridge et al. 2005; Lee et al. 2008); these patterns can also be sex-specific (Zajitschek et al. 2009; Zajitschek et al. 2012, 2013; Maklakov et al. 2009). Collectively, the extent to which diet can alter the expression of reproductive senescence is a topic that deserves further consideration, especially within the context of the entire life history. If, for example, diet alters egg production, diet may also alter the manner in which egg production deteriorates with age.

In this study, we employ a factorial experimental design and a freshwater snail system to explore the interactive effects of diet on the expression of adaptive plasticity in the age at first reproduction (i.e., delayed selfing), inbreeding depression, and reproductive senescence. As we are interested in the effects of diet on reproductive success, many of the patterns we explore are cross-generational, where the parental diet may affect the survival of offspring. Hence, maternal effects – effects that parents have on the phenotype of their offspring independent of offspring genotype (Bernardo 1996; Mousseau and Fox 1998; Donohue 2009) – may be involved. Under most conditions, diet significantly altered expression of these fitness-related traits.

## Methods

### Experimental design

A total of 578 snails from 22 full-sib families of *Physa acuta* were reared individually under two different diets and two mate-availability treatments. These experimental snails were the second-generation (G_2_) descendants of wild-caught (G_0_) adults from a pond near West Chester, PA. All G_0_ snails (*N* > 150) were isolated in the laboratory in 30-mL plastic boxes filled with carbon-filtered water; we collected eggs at 2-day intervals until we had ∼4 egg capsules from 100 adults. Three weeks later (1 week posthatching), G_1_ hatchlings were moved to fresh boxes with a constant density of 5 siblings/box. Water and food (boiled green leaf lettuce, provided *ad libitum*) were changed thrice/week. These hatchlings were isolated 3 weeks posthatching (shell length 2–3 mm), well before sexual maturity. This protocol promotes rapid development while ensuring virginity. At 9 weeks posthatching (shortly after maturity), individual G_1_ snails from different G_0_ mothers were paired; these snails served as parents for the full-sib G_2_ families. The same protocol of standardizing density was followed with G_2_ snails. When G_2_ snails were isolated, we split all snails from families with *N* > 20 into two diet treatments – 50% remained on a diet of boiled lettuce and 50% were switched to a diet of *Spirulina* flakes (O.S.I.; 41% crude protein [min], 4% crude fat [min], and 6% crude fiber [max]). Both diets were provided *ad libitum*; *Spirulina* is considered to be the higher quality diet. At 5 weeks posthatching (just prior to maturity), G_2_ snails in both diet treatments were split into two mate-availability treatments – 50% remained in isolation and 50% were provided with a mating partner. Mating partners came from extra families that were identically handled but not used in the experiment; these snails were marked with a harmless dot of paint (Henry and Jarne 2007). Mates were added to appropriate experimental containers for 3 h following each water change and feeding, a time period that is more than sufficient for copulation in both the male and female roles (Auld et al. 2014); containers were checked for eggs when the mates were removed and any eggs were removed because it is impossible to determine whether these eggs were laid by the focal individual or the mate. Once snails initiate egg laying, they typically lay an egg mass daily (Auld and Relyea 2008, 2010; J. R. Auld, pers. obs.). Placement of individuals in the laboratory was randomly assigned with respect to family and treatment and was re-arranged weekly.

For each individual snail, we recorded age and size at first reproduction (AFR and SFR, respectively) as well as reproductive success. During water changes, we checked for reproduction and recorded the date and size (maximum shell length) at which reproduction was initiated. We collected the first two egg capsules laid by each individual, counted these eggs, and set them aside to quantify hatching success of the G_3_ snails; adult snails were placed into a new box. Boiled lettuce was added to all boxes of eggs 5 day after eggs were isolated; hatching typically occurs in 7–10 day. We counted the number of unhatched eggs and surviving juveniles 15 day after eggs were laid – a time frame commonly used to assess both the hatching rate and immediate posthatching survival of juveniles (Escobar et al. 2007; Auld 2010; Auld et al. 2014). Snails with access to a partner lay 100% outcrossed eggs (or none at all; Pélissié et al. 2012), while isolated snails are forced to reproduce via self-fertilization (Jarne et al. 2000; Tsitrone et al. 2003b). Therefore, by comparing the reproductive success of isolated (selfing) and mated (outcrossing) snails, we can quantify inbreeding depression (see below). Egg-laying dates were converted to ages to quantify AFR and explore the effects of age on reproductive success (i.e., senescence).

### Statistical analyses

We fit generalized linear mixed models using the *glmer* command in R (*lme4* package; R Core Team 2012) that included the effects of diet, mate availability, and their interaction as fixed effects; family and its interaction with diet and mate availability were included as random effects. AFR and SFR were modeled as Gaussian variables. The survival rate of offspring (i.e., number of juveniles alive at 15 day/number of eggs) was modeled as a binomial variable (with logit link function) and was decomposed into hatching success and posthatching survival. That is, we modeled treatment effects on: (1) the entire early survival rate (i.e., # juveniles/# eggs), (2) the hatching rate (# hatchlings/# eggs), and (3) the posthatching survival rate (# juveniles/# hatchlings). The statistical significance of each term was determined using likelihood ratio tests comparing models with and without a given term (Bolker 2008). Models were compared using the chi-squared (*χ*^2^) test statistic. We tested the significance of family-by-treatment interactions by deleting these interactions one at a time from the full model. The family-by-treatment interactions were used to assess among-family variation in treatment effects (i.e., GxE interactions; Lynch and Walsh 1998). Further, the diet-by-mate interaction was used to determine whether the effects of mate availability (e.g., waiting time, inbreeding depression; see next paragraph) were affected by diet. These analyses were conducted (1) on the entire dataset and (2) after dropping individuals that failed to reproduce (*N* = 245). The conclusions were essentially identical – the same terms were always statistically significant; we report the results from analysis of the full dataset (including cases of no reproduction). Lastly, we explored the effects of clutch size (i.e., average number of eggs per egg mass) on the survival rate of offspring, particularly the hatching rate, by including clutch size as a covariate in the models.

The effect of mate availability on AFR reflects a change in the initiation of reproduction based on the availability of sexual partners. Hence, a significant mate effect on AFR corresponds to the presence of a waiting time (delay) before selfing. Comparatively, the effect of mates on the early survival of offspring corresponds to the presence of inbreeding depression. As previously stated, snails in the mate-available treatment reproduce via outcrossing while isolated snails are forced to self-fertilize; therefore any mate-associated difference in G_3_ early survival reflects a difference in G_2_ mating system. The waiting time before selfing (WT) and inbreeding depression (ID) were calculated separately for each family under each diet. WT was calculated as WT_*i*_ = AFR_*i*,NM_ − AFR_*i*,M_, where AFR was averaged over all individuals in the *i*^th^ family in the no-mate (NM) and mate (M) treatments. ID was calculated as 1 − (*W*_s_/*W*_o_) for eggs, hatchlings, and juveniles, where *W*_s_ is the number of offspring produced by isolated (selfing) snails and *W*_o_ is the corresponding number for mated (outcrossing) snails. Family-level ID values were compared to corresponding values for the waiting time by linear regression and Spearman's correlation coefficients. To test for heterogeneity of slopes in the relationship of WT and ID across diet treatments, we fit a model of WT including a diet-by-ID interaction.

As reproductive success was measured on each individual at multiple points in time, we also examined the effects of maternal age on offspring survival. A negative effect of age indicates the presence of reproductive senescence (Auld et al. 2014). We determined whether parental mating system and diet affected these age effects by evaluating the significance of the two-way interaction terms (i.e., age*mate availability and age*diet). That is, we tested whether parental mating system and diet affected the rate of reproductive senescence. As each individual reproduced multiple times, these models included individual identity as a random factor (i.e., a repeated-measures analysis). We evaluated the effects of age and age-by-treatment interactions on the hatching rate and posthatching survival of G_3_ snails.

## Results

**Figure 1:**
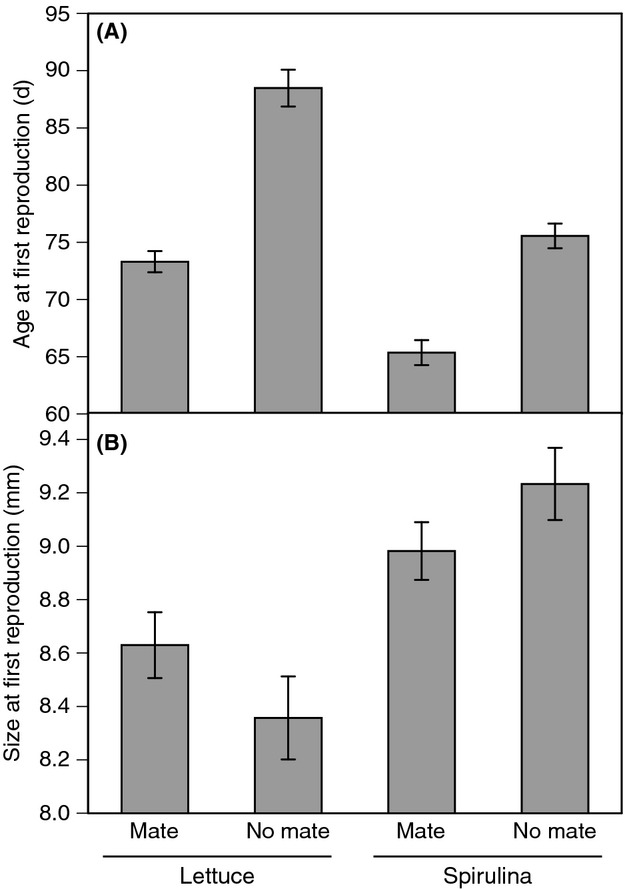
Average age (A) and size (B) at first reproduction in *Physa acuta* reared under two diet treatments and two conditions of mate availability. Error bars are ± 1 SE.

**Figure 2:**
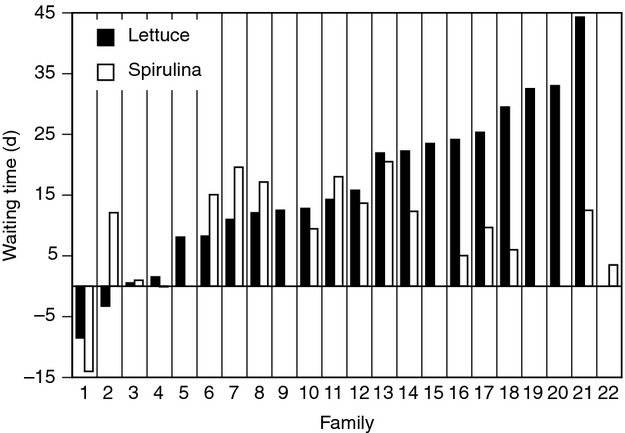
Waiting time (WT) before selfing of 22 full-sib families of *Physa acuta* reared on two diets. WT is defined (see text) as AFR_NM_ – AFR_M_ and reflects how long individuals delay self-fertilization in the absence of mates. Missing values reflect no reproduction in the no-mate treatment. Families are arranged in order of increasing WT when fed lettuce; numbers can be used to refer to Table S1. Sample size per family ranged from 3 to 32 with an average of 15.1 (SD = 7.8).

Age at first reproduction was significantly affected by diet, mate availability, and family (Table 1). Isolated snails delayed reproduction compared with mated snails (i.e., a waiting time before selfing; Figs. 1A and 2). Snails fed *Spirulina* initiated reproduction earlier than snails fed lettuce, and the magnitude of the waiting time was shorter under *Spirulina* (10.2 day) than lettuce (15.2 day). This difference in waiting time is reflected by the diet*mate interaction, which was borderline insignificant (*P* = 0.054; Table 1). The waiting time of lettuce-fed snails was not correlated with waiting time of *Spirulina*-fed snails (Spearman's *ρ* = 0.176, *P* = 0.512).

**Table 1:** Results of likelihood ratio tests comparing linear mixed models to assess the effects of diet, mate availability, family, and their two-way interactions on the age and size at first reproduction (AFR and SFR, respectively). Diet and mate availability (and their interaction) were analyzed as fixed effects, while family and interactions between family and fixed effects were analyzed as random effects. Models were constructed using the *glmer* command in R (*lme4* package), see text for further details

Trait	Factor	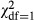	*P*
AFR	Diet	59.64	<0.001
	Mate	87.42	<0.001
	Diet*Mate	3.71	0.054
	Family	19.96	<0.001
	Family*Diet	0	1
	Family*Mate	0.86	0.354
SFR	Diet	19.75	<0.001
	Mate	0.06	0.809
	Diet*Mate	3.67	0.055
	Family	31.61	<0.001
	Family*Diet	0	1
	Family*Mate	0.17	0.678

Snails fed *Spirulina* were also larger at first reproduction than snails fed lettuce (Fig. 1B). There was no significant mate effect on SFR, although the diet*mate interaction (*P* = 0.055) indicated that isolated snails tended to be larger than mated snails if fed *Spirulina*, while the converse pattern was observed for lettuce-fed snails.

**Figure 3:**
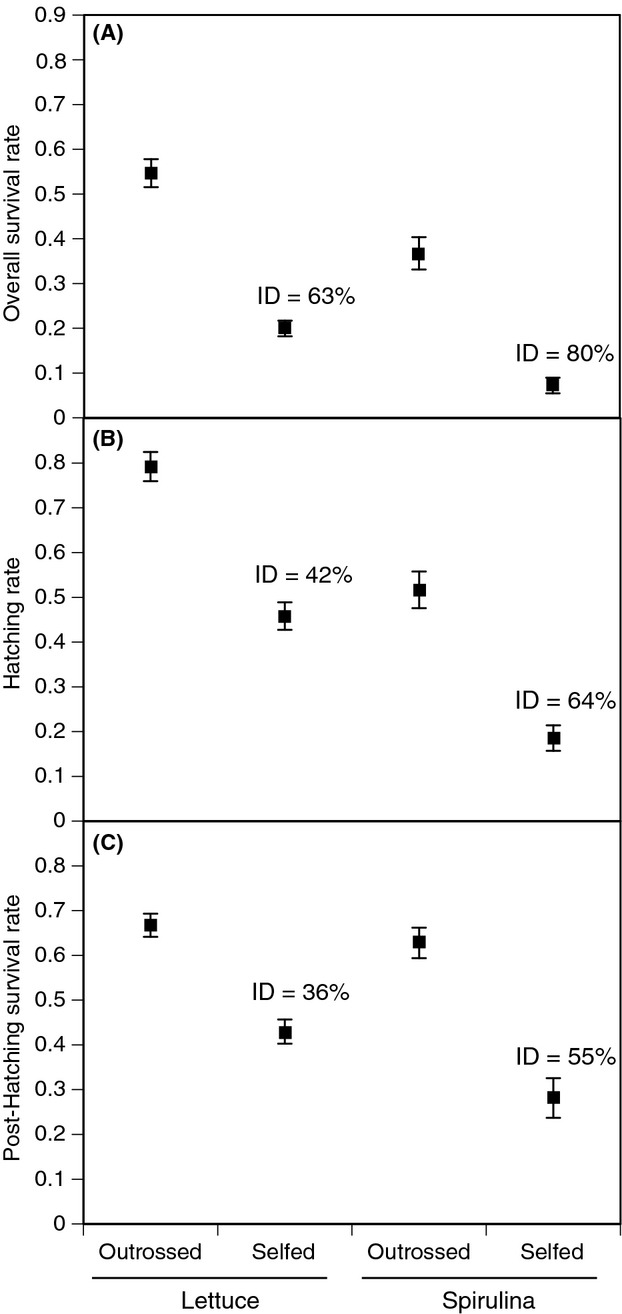
Overall survival rate (A; #juveniles alive at 15 day/#eggs), hatching rate (B; #hatchlings/#eggs), and posthatching survival rate (C; #juveniles alive at 15 day/#hatchlings) of *Physa acuta* produced through outcrossing (parents with mates) or selfing (parents without mates). Parental snails were fed either lettuce or Spirulina; juvenile snails were fed lettuce. Inbreeding depression (ID) values are inset (see text). Error bars are ± 1 SE.

The early survival of eggs and juvenile snails was also affected by diet, mating system, and family (Table 2; Fig. 3A). Here, it is crucial to remember that treatments were applied to G_2_ snails (parents), but we assessed the survival of G_3_ snails (offspring). The overall survival rate of offspring (Table 2A) was variable among families, including variation among families in the effects of diet and parental mating system (family-by-treatment interactions). On average, offspring of isolated parents had much lower survival than offspring of mated parents (i.e., inbreeding depression), but the magnitude of inbreeding depression was significantly larger when parents were fed *Spirulina* (80.3%; mean comparison, *F*_1,149_ = 59.2, *P* < 0.001) compared with lettuce (63.5%; *F*_1,180_ = 86.3, *P* < 0.001). Family-level means (±SE) of eggs, hatchlings, and juveniles are in Table S1.

**Table 2:** Results of likelihood ratio tests comparing generalized linear mixed models fit to assess the effects of diet, mate availability (mating system), family, and their two-way interactions on the survival of offspring. We tested treatment effects on (A) the overall early survival rate of juveniles controlling for the number of eggs (i.e., Eggs → Juveniles). This overall survival rate was then decomposed (B) into the hatching rate (i.e., Eggs → Hatchlings) and the posthatching survival rate (i.e., Hatchlings → Juveniles). Terms were analyzed and calculated as in Table 1

Fitness Component	Factor	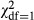	*P*
(A)			
Early Survival (Egg → Juvenile)	Diet	1353	<0.001
	Mate	3724	<0.001
	Diet*Mate	104	<0.001
	Family	1614	<0.001
	Family*Diet	1218	<0.001
	Family*Mate	440	<0.001
(B)			
Hatching Rate (Egg → Hatchling)	Diet	3202	<0.001
	Mate	4191	<0.001
	Diet*Mate	0.59	0.441
	Family	1792	<0.001
	Family*Diet	1530	<0.001
	Family*Mate	756	<0.001
Posthatching Survival (Hatchling → Juvenile)	Diet	5.98	0.014
	Mate	663	<0.001
	Diet*Mate	29.1	<0.001
	Family	545	<0.001
	Family*Diet	332	<0.001
	Family*Mate	90.1	<0.001

**Figure 4:**
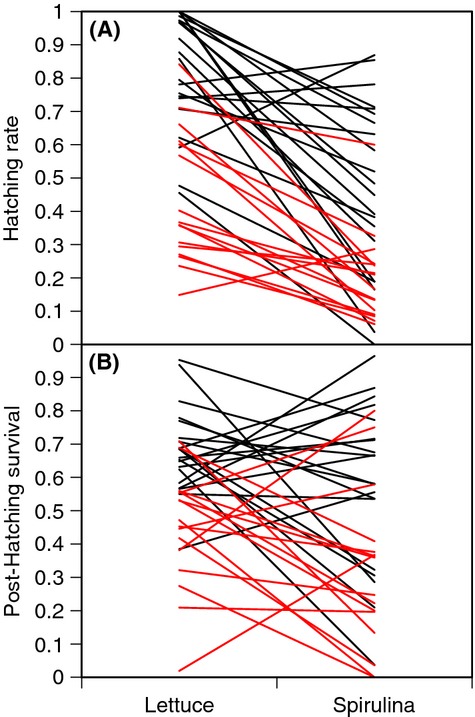
Family-by-treatment interactions showing the change in (A) hatching rate and (B) posthatching survival for each family across lettuce and *Spirulina* treatments, black lines are from the mated (outcrossed) treatment, while red lines are from the isolated (selfed) treatment.

Decomposing the early survival of offspring into its two components (i.e., egg hatching and posthatching survival of juveniles) reveals that the effects of parental diet are expressed in both stages (Table 2B, Fig. 3), although they are slightly more pronounced for hatching rate (*cf*. Fig. 3B,C). Also, the diet-by-mate interaction was only significant for posthatching survival rate indicating that the diet-induced change in inbreeding depression primarily affects posthatching survival. There was significant among-family variation in the effects of diet for both egg hatching and posthatching survival (family*diet interaction) and significant among-family variation in the magnitude of inbreeding depression (family*mate interaction). These interactions can be visualized in Figure 4. Clutch size (the average number of eggs per mass) was not a significant covariate in the models of early survival or hatching rate (results not shown).

**Figure 5:**
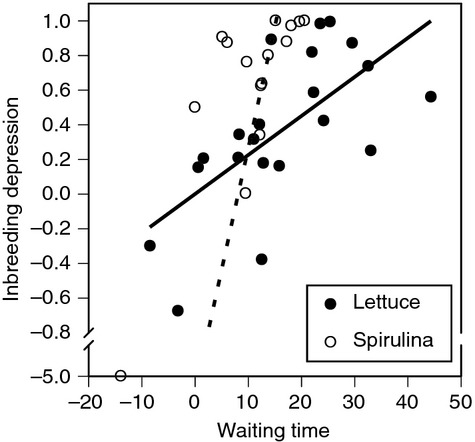
Family-level inbreeding depression (based on the number of surviving juveniles) plotted against family-level waiting time (i.e., the delay in selfing in the absence of mates) for *Physa acuta* snails fed lettuce (solid symbols, solid line) or Spirulina (open symbols, dashed line). Waiting time is calculated as AFR_NM_ – AFR_M_ (see text), so negative values indicate prior selfing. Inbreeding depression is calculated as 1-(Ws/Wo), where Ws is the number of surviving juveniles from the no mate-available treatment and Wo is the corresponding value from the mate-available treatment (see text). An axis break is used on the *y*-axis.

A linear regression of family-level waiting time on family-level inbreeding depression based on the number of surviving juveniles revealed a significant, positive relationship under lettuce (*r*^2^ = 0.40, *P* = 0.001) and *Spirulina* (*r*^2^ = 0.64, *P* < 0.001). These relationships are plotted in Figure 5. Spearman's correlation coefficients were also statistically significant (lettuce: *ρ* = 0.677, *P* = 0.001; *Spirulina*: *ρ* = 0.718, *P* = 0.002). Furthermore, the slopes were significantly different (diet-by-ID interaction: *F*_1,34_ = 10.7, *P* = 0.003), indicating that diet alters the relationship between waiting time and inbreeding depression.

**Figure 6:**
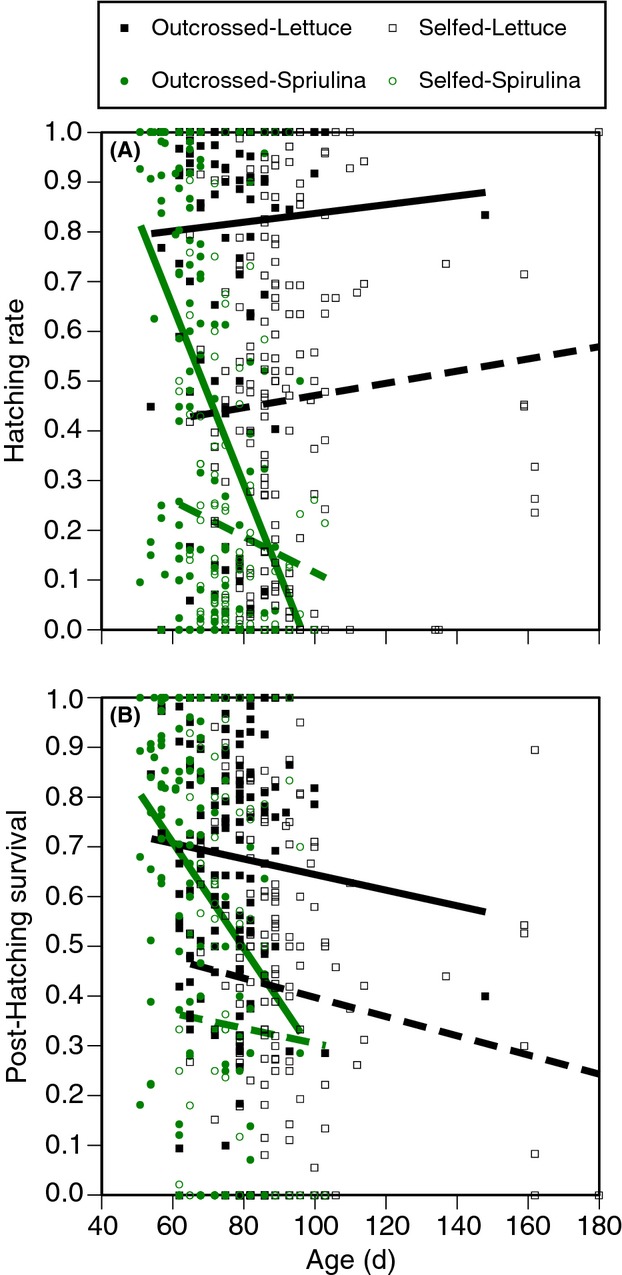
The effects of maternal age on the (A) hatching rate and (B) posthatching survival. Mated snails reproduced via *outcrossing* (closed symbols) while isolated snails reproduced via *selfing* (open symbols). *Lettuce* (black) and *Spirulina* (green) refer to maternal diets. Lines are linear regressions.

Overall, there was a significant, negative effect of maternal age on both egg hatching and posthatching survival, but this effect depended on parental diet and mating system (Table 3, Fig. 6). Aside from the reduction in hatching rate and posthatching survival associated with *Spirulina* (Table 2, Figs. 3, 4), *Spirulina* also accelerated the rate of reproductive senescence – an effect that was particularly strong for outcrossed snails. A diet of lettuce also resulted in reproductive senescence, but the effects were primarily restricted to posthatching survival and not hatching rate.

**Table 3:** Results of likelihood ratio tests comparing generalized linear mixed models fit to assess the effects of maternal age and its interaction with mate availability (mating system) and diet on the hatching rate and posthatching survival. The models include individual identity as a random effect (i.e., a repeated-measures analysis)

Fitness Component	Factor	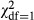	*P*
Hatching Rate (Egg → Hatchling)	Age	47.2	<0.001
	Age*Mate	56.2	<0.001
	Age*Diet	123.5	<0.001
Posthatching Survival (Hatchling → Juvenile)	Age	29.3	<0.001
	Age*Mate	8.09	0.004
	Age*Diet	29.8	<0.001

## Discussion

Relative to lettuce, a diet of *Spirulina* had a number of substantial effects. Snails that were fed *Spirulina* reproduced earlier, and at a larger size, than snails that were fed lettuce. *Spirulina* also reduced the average waiting time before selfing by 30%. Inbreeding depression in offspring survival was apparent, strong, and significantly affected by parental diet. Snails that were fed *Spirulina* experienced reduced offspring survival, an effect that can be attributed to a reduction in hatching success. Furthermore, there were negative effects of parental age on offspring survival, and importantly, these effects were much stronger when snails were fed *Spirulina* than lettuce. Genetic variation was present for all traits that were studied, and among-family variation in treatment effects (i.e., GxE interactions) was observed for reproductive success. Below we discuss our main results in turn, focusing primarily on the manner in which diet alters the expression of delayed selfing, inbreeding depression, and reproductive senescence.

The effects of diet on life history (age at first reproduction) expression shed light on previous discrepancies in the estimation of the waiting time before selfing in this species. As expected (Tsitrone et al. 2003a), self-compatible, preferentially outcrossing hermaphrodites delayed selfing when mates were absent. This has been repeatedly observed in this species and others (Escobar et al. 2011; Ramm et al. 2012). However, previous estimates of the magnitude of the delay in selfing in *Physa acuta* range from a few days (Auld and Relyea 2008), to a few weeks (e.g., Tsitrone et al. 2003b; Escobar et al. 2011), to >2 months (Auld 2010). While among-population variation in the waiting time is certainly a contributing factor (Escobar et al. 2009), the diet of the snails is an additional factor that can explain some of this variation. Most studies have used lettuce as food, but those yielding shorter estimates of the waiting time (Auld and Relyea 2008; this study) have fed the snails *Spirulina*. This represents an important issue for consideration when extrapolating experimental results to natural systems – we do not have estimates of the waiting time under natural (wild) conditions and while these snails are generalist grazers on periphyton, the realized waiting time in a natural system is certainly likely to be affected by the local resources available.

This study not only confirms the existence of the waiting time but also confirms a theoretically predicted positive relationship between the waiting time and the magnitude of inbreeding depression (Tsitrone et al. 2003a; Escobar et al. 2011). Importantly, the relationship observed here is based on intrapopulation, among-family variation such that families with longer waiting times experience stronger inbreeding depression. This is particularly interesting for at least two reasons. First, this relationship is predicted to exist at the among-population level based on the correlation between inbreeding depression and the evolution of the selfing (outcrossing) rate (Lande and Schemske 1985; Crnokrak and Barrett 2002), but at the within-population level, this relationship is expected to be very short-lived (Schultz and Willis 1995). This is not the first time this relationship has been observed at this level in this species (Escobar et al. 2009; Auld 2010) or other species (Vogler et al. 1999; Takebayashi and Delph 2000; Stone and Motten 2002), but other studies on this species have reported it to be nonexistent (Escobar et al. 2007) or only apparent under particular environmental conditions (Auld 2010). The extent to which the genetic architecture of these traits changes under different environmental conditions is not known. Second, while the delay in selfing and the magnitude of inbreeding depression are significantly correlated under both diet treatments, this is not because the same families exhibit long waiting times and strong inbreeding depression in both environments. As evidenced by Figure 2 (and Table S1), the waiting time before selfing for lettuce-fed snails is a poor predictor of the waiting time in *Spirulina*-fed snails. The relationship between waiting time and inbreeding depression exists in both environments but is apparently independent in those two environments.

This study adds to the growing body of literature indicating that inbreeding depression is often environment-specific (Armbruster and Reed 2005; Cheptou and Donohue 2011). However, the effects that we observed are not a result of inbred and outbred individuals being exposed to divergent environmental conditions, these are transgenerational effects. It has been previously observed in this species that parental exposure to predator cues can reduce inbreeding depression (Auld 2010). Here, parental exposure to a lower quality diet reduced inbreeding depression. The effects of parental diet on offspring survival were most pronounced during egg hatching. While *Spirulina* had overall positive effects on adult growth, leading to an acceleration of the age at first reproduction; it had clear negative consequences for offspring survival.

While the mechanism(s) underlying environment-dependent inbreeding depression are not well understood (Cheptou and Donohue 2011), Waller et al. (2008) proposed that the change in inbreeding depression across environments may be related to Crow's “opportunity for selection” (*I*; Waller et al. 2008), defined as the squared coefficient of phenotypic variation. Relative to a diet of lettuce, *Spirulina* increased this value twofold for mated snails and fourfold for isolated snails (results not shown). As such, our data support Waller et al.'s (2008) contention that inbreeding depression should be higher in the environment where the opportunity for selection is higher.

The reduction in hatching success of eggs laid by *Spirulina*-fed parents could have a number of explanations. First of all, we can rule out an effect of density – the number of eggs per mass did not differ based on adult diet, and our analyses indicated no relationship between the number of eggs and the hatching rate of those eggs. Second, there could be some micro-environmental effects (e.g., in the laboratory environment) that differed between when the *Spirulina*-fed snails were reproducing and when the lettuce-fed snails were reproducing. There were no noticeable changes in the laboratory, and this seems to be an unlikely explanation as the mated, lettuce-fed snails were reproducing concurrently with the isolated, *Spirulina*-fed snails (Fig. 1A), but the difference in hatching success is extreme (Fig. 3B). Third, a trade-off could exist between age at first reproduction and hatching success in the next generation. It is possible that initiating reproduction at too early an age may be detrimental to the survival of offspring. Such an effect could stem from the parental snails inability to produce high quality eggs. Fourth, there may be a pure transgeneration effect of food quality. *Spirulina* may have negative effects on offspring survival despite positive effects on adult growth if, for example, something toxic is present in the *Spirulina*. At present, we have no way to evaluate the fourth hypothesis, whereas the third (trade-off) seems to warrant future evaluation.

Parental age had a direct, negative consequence on offspring survival – as the snails aged, the survival of their offspring dropped. This effect was variable among families, stronger for mated (outcrossing) snails, and much stronger if the parents were fed *Spirulina* rather than lettuce. Considering Figure 6, the effect of mate availability (mating system) may perhaps be an artifact of the fact that selfing snails had an overall lower offspring survival rate than outcrossing snails (i.e., inbreeding depression). However, the difference in the age-related decline in offspring survival between lettuce-fed snails and *Spirulina*-fed snails is striking. While *Spirulina* is clearly beneficial in terms of increasing individual growth and reducing generation time, its effects on offspring survival and the rate of senescence are overwhelmingly negative. This result is consistent with previous research (e.g., Moya-Laraño 2002; Partridge et al. 2005; Lee et al. 2008) in the sense that an overabundance of resources can result in an acceleration of senescence. Future research is needed to explore the optimization of survival and reproductive success at intermediate resource levels and distinguish the role of individual nutrients (e.g., carbohydrates, protein, etc.).

In conclusion, diet altered numerous aspects of individual life history and mating-system expression with important transgenerational consequences. As we extend our understanding of this system and attempt to generalize laboratory studies into natural systems, it is extremely important to consider the ripple effects that something such as diet may have on a variety of fitness-related traits. Future work is needed to understand the expression of these traits under natural conditions, and particularly how variation in natural food availability alters trait expression that can have implications on numerous other traits and population-level dynamics such as the maintenance of genetic variation.

## Supporting Information

**Table S1.** Family-level means (SE) for the number of outcrossed (O) or selfed (S) G_3_ *Physa acuta* eggs, hatchlings and juveniles (alive at 15 day) produced by parents fed either lettuce or *Spirulina*.

## References

[b1] Armbruster P, Reed DH (2005). Inbreeding depression in benign and stressful environments. Heredity.

[b2] Auld JR (2010). The effects of predation risk on mating system expression in a freshwater snail. Evolution.

[b3] Auld JR, Relyea RA (2008). Are there interactive effects of mate availability and predation risk on life history and defense in a simultaneous hermaphrodite?. J. Evol. Biol.

[b4] Auld JR, Relyea RA (2010). Life history plasticity and inbreeding depression under mate limitation and predation risk: Cumulative lifetime fitness dissected with a life table response experiment. Evol. Ecol.

[b5] Auld JR, Jarne P, Sarda V, Jourdan-Pineau H, Lamy T, Pelissie B, David P (2014). Evaluating the contributions of change in investment and change in efficiency to age-related declines in male and female reproduction. J. Evol. Biol.

[b6] Bernardo J (1996). Maternal effects in animal ecology. Amer. Zool.

[b7] Bolker BM (2008). Ecological models and data in R.

[b9] Charlesworth D, Charlesworth B (1987). Inbreeding depression and its evolutionary consequences. Ann. Rev. Ecol. Syst.

[b10] Charmantier A, Perrins C, McCleery RH, Sheldon BC (2006). Quantitative genetics of age at reproduction in wild swans: Support for antagonistic pleiotropy models of senescence. Proc. Natl Acad. Sci. USA.

[b11] Cheptou P-O, Donohue K (2011). Environment-dependent inbreeding depression: Its ecological and evolutionary significance. New Phytol.

[b12] Chippindale AK, Leroi AM, Saing H, Borash DJ, Rose MR (1997). Phenotypic plasticity and selection in *Drosophila* life history evolution. II. Diet, mates and the cost of reproduction. J. Evol. Biol.

[b14] Crnokrak P, Barrett SCH (2002). Purging the genetic load: A review of the experimental evidence. Evolution.

[b15] Donohue K (2009). Completing the cycle: Maternal effects as the missing link in plant life histories. Philos. Trans. R. Soc. B.

[b16] Escobar JS, Epinat G, Sarda V, David P (2007). No correlation between inbreeding depression and delayed selfing in the freshwater snail *Physa acuta*. Evolution.

[b17] Escobar JS, Jarne P, Charmantier A, David P (2008). Outbreeding alleviates senescence in hermaphroditic snails as expected from the mutation-accumulation theory. Curr. Biol.

[b18] Escobar JS, Facon B, Jarne P, Goudet J, David P (2009). Concerted micro-evolution of mating strategy and inbreeding depression in hermaphroditic snails. Evolution.

[b19] Escobar JS, Auld JR, Correa AC, Alonso JM, Bony YK, Coutellec M-A (2011). Patterns of mating system evolution in hermaphroditic animals: Correlations among selfing rate, inbreeding depression, and the timing of reproduction. Evolution.

[b20] Harwood JF, Chen K, Muller H-G, Wang J-L, Vargas RI, Carey JR (2013). Effects of diet and host access on fecundity and lifespan in two fruit fly species with different life-history patterns. Physiol. Entomol.

[b21] Henry P-Y, Jarne P (2007). Marking hard-shelled gastropods: Tag loss, impact on life-history traits, and perspectives in biology. Invert. Biol.

[b22] Jarne P, Perdieu M-A, Pernot A-F, Delay B, David P (2000). The influence of self-fertilization and grouping on fitness attributes in the freshwater snail *Physa acuta*: Population and individual inbreeding depression. J. Evol. Biol.

[b23] Johnston MO, Schoen DJ (1994). On the measurement of inbreeding depression. Evolution.

[b24] Kirkwood TBL, Shanley DP (2005). Food restriction, evolution and ageing. Mech. Ageing Devel.

[b25] Lande R, Schemske DW (1985). The evolution of self-fertilization and inbreeding depression in plants. I. Genetic models. Evolution.

[b26] Lee KP, Simpson SJ, Clissold FJ, Brooks R, Balland JWO, Taylor PW (2008). Lifespan and reproduction in *Drosophila*: New insights from nutritional geometry. Proc. Natl. Acad. Sci. USA.

[b27] Lloyd DG (1979). Some reproductive factors affecting the selection of self-fertilization in plants. Am. Nat.

[b28] Lynch M, Walsh B (1998). Genetics and analysis of quantitative traits.

[b29] Maklakov AA, Hall MD, Simpson SJ, Dessmann J, Clissold FJ, Zajitscheck F (2009). Sex differences in nutrient-dependent reproductive ageing. Aging Cell.

[b30] Mousseau TA, Fox CW (1998). The adaptive significance of maternal effects. Trends Ecol. Evol.

[b31] Moya-Laraño J (2002). Senescence and food limitation in a slowly ageing spider. Func. Ecol.

[b32] van Noordwijk AJ, de Jong G (1986). Acquisition and allocation of resources: Their influence in variation in life-history tactics. Am. Nat.

[b33] Partridge L, Pletcher SD, Mair W (2005). Dietary restriction, mortality trajectories, risk and damage. Mech. Ageing Devel.

[b34] Pélissié B, Jarne P, David P (2012). Sexual selection without sexual dimorphism: Bateman gradients in a hermaphrodite. Evolution.

[b35] R Core Team (2012). R: A language and environment for statistical computing.

[b36] Ramm SA, Vizoso DB, Schärer L (2012). Occurrence, costs and heritability of delayed selfing in a free-living flatworm. J. Evol. Biol.

[b37] Roff DA (2002). Life history evolution.

[b38] Rose MR (1991). Evolutionary biology of aging.

[b39] Schultz ST, Willis JH (1995). Individual variation in inbreeding depression: The roles of inbreeding history and mutation. Genetics.

[b40] Shanley DP, Kirkwood TBL (2000). Calorie restriction and aging: A life-history analysis. Evolution.

[b41] Stearns SC (1992). The evolution of life histories.

[b42] Stone JL, Motten AF (2002). Anther-stigma separation is associated with inbreeding depression in *Datura stramonium*, a predominantly self-fertilization annual. Evolution.

[b43] Takebayashi N, Delph LF (2000). An association between a floral trait and inbreeding depression. Evolution.

[b44] Tsitrone A, Duperron S, David P (2003a). Delayed selfing as an optimal mating strategy in preferentially outcrossing species: Theoretical analysis of the optimal age at first reproduction in relation to mate availability. Am. Nat.

[b45] Tsitrone A, Jarne P, David P (2003b). Delayed selfing and resource reallocation in relation to mate availability in the freshwater snail *Physa acuta*. Am. Nat.

[b46] Vogler DW, Filmore K, Stephenson AG (1999). Inbreeding depression in *Campanula rapunculoides* L. I. A comparison of inbreeding depression in plants derived from strong and weak self-incompatibility phenotypes. J. Evol. Biol.

[b47] Waller DM, Dole J, Bersch AJ (2008). Effects of stress and phenotypic variation on inbreeding depression in *Brassica rapa*. Evolution.

[b48] Zajitschek F, Hunt J, Jennions MD, Hall MD, Brooks RC (2009). Effects of juvenile and adult diet on ageing and reproductive effort of male and female black field crickets, *Teleogryllus commodus*. Func. Ecol.

[b49] Zajitschek F, Lailvaux SP, Dessmann J, Brooks R (2012). Diet, sex, and death in field crickets. Ecol. Evol.

[b50] Zajitschek F, Zajitschek SRK, Friberg U, Maklakov AA (2013). Interactive effects of sex, social environment, dietary restriction, and methionine on survival and reproduction in fruit flies. Age.

