# Supplementary material for: Diet alters delayed selfing, inbreeding depression, and reproductive senescence in a freshwater snail
Source: Ecol Evol. 2014 Jun 22;4(14):2968–77. doi: 10.1002/ece3.1146 (PMC4130452; doi:10.1002/ece3.1146)
Supplement: Supplementary file 1 — Table S1. Family-level means (SE) for the number of outcrossed (O) or selfed (S) G3 Physa acuta eggs, hatchlings and juveniles (alive at 15 day) produced by parents fed either lettuce or Spirulina. [file ece30004-2968-SD1.doc]

**Table S1:** Family-level means (S.E.) for the number of outcrossed (O) or selfed (S) G_3_ *Physa acuta* eggs, hatchlings and juveniles (alive at 15d) produced by parents fed either lettuce or *Spirulina*. N.B. diet treatments were applied in the parental (G_2_) generation; all hatchling/juvenile snails were fed lettuce. Inbreeding depression (ID) values are given for each family/diet treatment combination. Family numbers correspond with Fig. 2. Grand means (S.E.) are also listed.

|  | Eggs | | | | | | Hatchlings | | | | | | Juveniles | | | | | |
| --- | --- | --- | --- | --- | --- | --- | --- | --- | --- | --- | --- | --- | --- | --- | --- | --- | --- | --- |
|  | Lettuce | | | Spirulina | | | Lettuce | | | Spirulina | | | Lettuce | | | Spirulina | | |
| **Family** | **O** | **S** | **ID** | **O** | **S** | **ID** | **O** | **S** | **ID** | **O** | **S** | **ID** | **O** | **S** | **ID** | **O** | **S** | **ID** |
| 1 | 3.2  (3.2) | 25.2  (16.4) | -6.86 | 7.4  (7.4) | 3.6  (3.6) | 0.52 | 3.2  (3.2) | 17.8  (11.5) | -4.57 | 1.4  (1.4) | 2.1  (2.1) | -0.53 | 3.0  (3.0) | 6.5  (4.1) | -1.17 | 0.4  (0.4) | 1.7  (1.7) | -3.29 |
| 2 | 4.2  (4.2) | 72.0  (24.9) | -16.14 | 76.5  (12.3) | 103.0  (27.3) | -0.35 | 4.2  (4.2) | 39.0  (16.1) | -8.29 | 49.2  (12.5) | 28.6  (13.7) | 0.42 | 4.0  (4.0) | 26.8  (11.3) | -5.70 | 39.8  (12.0) | 23.0  (11.2) | 0.42 |
| 3 | 20.2  (6.7) | 80.3  (24.8) | -2.98 | 17.8  (9.0) | 26.3  (26.3) | -0.48 | 13.3  (7.3) | 17.6  (4.6) | -0.32 | 6.2  (5.5) | 6.3  (6.3) | -0.02 | 10.0  (6.3) | 8.7  (3.3) | 0.13 | 5.8  (5.1) | 3.7  (3.7) | 0.37 |
| 4 | 35.0  (12.8) | 38.5  (16.9) | -0.10 | 44.0  (27.9) | 46.5  (21.9) | -0.06 | 35.0  (12.8) | 21.1  (10.8) | 0.40 | 5.7  (4.7) | 5.2  (3.5) | 0.09 | 26.8  (9.7) | 13.3  (7.1) | 0.50 | 4.0  (3.6) | 1.3  (1.3) | 0.67 |
| 5 | 58.8  (18.8) | 80.0  (38.5) | -0.36 | 54.8  (14.7) | 0  (.) | 1.0 | 46.8  (11.9) | 59.2  (30.1) | -0.26 | 2.3  (0.9) | 0  (.) | 1.0 | 30.0  (8.0) | 17.8  (9.7) | 0.41 | 0.8  (0.5) | 0  (.) | 1.0 |
| 6 | 64.5  (16.8) | 99.9  (32.1) | -0.55 | 62.0  (22.1) | 24.2  (21.4) | 0.61 | 51.8  (18.7) | 56.0  (15.4) | -0.08 | 50.8  (23.0) | 0.1  (0.1) | 1.0 | 39.7  (14.2) | 31.3  (10.4) | 0.21 | 32.8  (15.1) | 0  (.) | 1.0 |
| 7 | 58.5  (9.1) | 61.4  (18.7) | -0.05 | 112.8  (36.7) | 48.6  (26.2) | 0.57 | 57.0  (8.9) | 39.3  (16.2) | 0.31 | 42.2  (11.9) | 2.0  (0.9) | 0.95 | 37.5  (3.6) | 17.1  (5.5) | 0.54 | 27.7  (11.3) | 0.1  (0.1) | 1.0 |
| 8 | 48.8  (17.4) | 46.2  (27.2) | 0.05 | 82.8  (16.2) | 52.5  (33.0) | 0.37 | 48.2  (17.2) | 29.2  (17.2) | 0.39 | 64.0  (18.9) | 10.7  (5.3) | 0.83 | 33.0  (12.0) | 14.8  (9.2) | 0.55 | 52.3  (13.3) | 3.2  (2.1) | 0.94 |
| 9 | 33.7  (21.5) | 37.4  (37.4) | -0.11 | 11.5  (11.5) | 0  (.) | 1.0 | 26.0  (17.7) | 24.9  (24.9) | 0.04 | 4.5  (4.5) | 0  (.) | 1.0 | 17.2  (12.6) | 10.1  (10.1) | 0.41 | 0.2  (0.2) | 0  (.) | 1.0 |
| 10 | 77.2  (28.0) | 108.9  (20.6) | -0.41 | 46.7  (13.4) | 85.9  (23.2) | -0.84 | 73.8  (26.5) | 92.8  (20.1) | -0.26 | 19.7  (6.2) | 21.4  (10.6) | -0.09 | 51.2  (17.8) | 52.6  (11.6) | -0.03 | 13.2  (5.7) | 10.5  (5.3) | 0.20 |
| 11 | 93.5  (14.5) | 58.0  (18.8) | 0.38 | 75.3  (28.7) | 67.6  (26.3) | 0.10 | 84.3  (15.0) | 13.7  (6.6) | 0.84 | 67.3  (26.7) | 8.3  (5.2) | 0.88 | 60.7  (15.9) | 4.4  (2.7) | 0.93 | 53.0  (19.7) | 1.1  (0.8) | 0.98 |
| 12 | 45.2  (11.4) | 64.6  (35.5) | -0.43 | 85.5  (25.1) | 60.0  (25.4) | 0.30 | 32.8  (14.7) | 24.2  (15.8) | 0.26 | 47.3  (14.1) | 7.7  (4.7) | 0.84 | 25.3  (11.4) | 12.8  (9.1) | 0.50 | 31.5  (10.2) | 3.7  (3.4) | 0.88 |
| 13 | 66.7  (13.9) | 46.3  (17.2) | 0.31 | 52.8  (15.0) | 12.6  (10.3) | 0.76 | 45.7  (10.5) | 11.3  (4.3) | 0.75 | 38.5  (15.2) | 1.3  (1.3) | 0.97 | 24.8  (5.7) | 3.2  (1.7) | 0.87 | 33.3  (13.8) | 0  (.) | 1.0 |
| 14 | 66.8  (20.2) | 91.0  (25.5) | -0.36 | 124.4  (50.9) | 47.3  (34.9) | 0.62 | 60.8  (17.8) | 26.8  (14.1) | 0.56 | 20.6  (9.8) | 3.0  (2.0) | 0.85 | 43.4  (16.7) | 18.0  (9.1) | 0.59 | 3.2  (2.0) | 0.5  (0.5) | 0.84 |
| 15 | 37.3  (20.9) | 8.8  (7.8) | 0.77 | 35.8  (18.1) | 0  (.) | 1.0 | 27.0  (23.1) | 2.3  (1.9) | 0.92 | 15.0  (7.4) | 0  (.) | 1.0 | 21.3  (20.3) | 0.3  (0.3) | 0.99 | 8.0  (3.8) | 0  (.) | 1.0 |
| 16 | 77.5  (12.6) | 92.3  (11.4) | -0.19 | 95.7  (11.4) | 112.9  (30.7) | -0.18 | 45.7  (8.5) | 29.2  (8.1) | 0.36 | 42.0  (16.9) | 25.5  (12.5) | 0.39 | 22.7  (4.7) | 12.0  (3.8) | 0.47 | 20.0  (6.1) | 1.4  (1.2) | 0.93 |
| 17 | 82.0  (26.3) | 38.2  (23.4) | 0.53 | 91.2  (52.3) | 88.4  (42.2) | 0.03 | 59.4  (19.5) | 6.8  (5.0) | 0.89 | 83.4  (50.7) | 22.6  (15.9) | 0.73 | 41.2  (17.2) | 0.2  (0.2) | 1.0 | 68.8  (47.2) | 16.4  (15.4) | 0.76 |
| 18 | 26.4  (26.4) | 80.0  (28.2) | -2.03 | 84.0  (38.4) | 109.4  (34.2) | -0.30 | 26.4  (26.4) | 22.8  (9.9) | 0.14 | 61.0  (21.6) | 13.2  (5.5) | 0.78 | 17.4  (17.4) | 9.0  (2.6) | 0.48 | 42.8  (15.0) | 5.4  (3.7) | 0.87 |
| 19 | 61.4  (21.3) | 57.0  (24.6) | 0.07 | 65.6  (16.6) | 0  (.) | 1.0 | 48.0  (19.0) | 23.2  (13.5) | 0.52 | 22.0  (7.0) | 0  (.) | 1.0 | 33.6  (16.4) | 8.8  (5.4) | 0.74 | 7.2  (3.3) | 0  (.) | 1.0 |
| 20 | 20.0  (14.9) | 45.4  (33.8) | -1.27 | 0  (.) | 0  (.) |  | 19.6  (14.7) | 26.0  (19.8) | -0.33 | 0  (.) | 0  (.) |  | 16.0  (11.5) | 18.0  (15.4) | -0.13 | 0  (.) | 0  (.) |  |
| 21 | 65.0  (5.3) | 28.0  (18.0) | 0.57 | 19.6  (12.0) | 28.3  (18.4) | -0.44 | 50.4  (10.0) | 6.8  (4.8) | 0.86 | 8.6  (5.7) | 2.3  (2.3) | 0.73 | 28.4  (7.0) | 4.2  (3.3) | 0.85 | 6.6  (5.4) | 1.7  (1.7) | 0.74 |
| 22 | 0  (.) | 0  (.) |  | 13.6  (9.6) | 16.0  (16.0) | -0.18 | 0  (.) | 0  (.) |  | 0  (.) | 0  (.) |  | 0  (.) | 0  (.) |  | 0  (.) | 0  (.) |  |
|  | | | | | | | | | | | | | | | | | | |
| **MEAN** | 48.4  (4.0) | 60.5  (5.5) | -0.25 | 58.4  (5.7) | 47.1  (5.8) | 0.19 | 39.9  (3.6) | 28.7  (3.4) | 0.28 | 30.9  (4.0) | 8.1  (1.6) | 0.74 | 27.2  (2.7) | 14.2  (1.9) | 0.48 | 21.5  (3.2) | 3.3  (0.9) | 0.85 |
